# Mutational analysis on predicting the impact of high-risk SNPs in human secretary phospholipase A2 receptor (PLA2R1)

**DOI:** 10.1038/s41598-020-68696-7

**Published:** 2020-07-16

**Authors:** Zoya Khalid, Omar Almaghrabi

**Affiliations:** 1 Computational Biology Research Lab, Department of Computer Science, National University of Computing and Emerging Sciences, NUCES-FAST, Islamabad, Pakistan; 2 Department of Biology, College of Science, University of Jeddah, Jeddah, Saudi Arabia

**Keywords:** Computational platforms and environments, Data mining, Computational biology and bioinformatics, Diseases

## Abstract

PLA2R1 is a transmembrane glycoprotein that acts as an endogenous ligand which stimulates the processes including cell proliferation and cell migration. The SNPs in PLA2R1 is associated with idiopathic membranous nephropathy which is an autoimmune kidney disorder. The present study aimed to explore the structure–function analysis of high risk SNPs in PLA2R1 by using 12 different computational tools. First the functional annotation of SNPs were carried out by sequence based tools which were further subjected to evolutionary conservation analysis. Those SNPs which were predicted as deleterious in both categories were further considered for structure based analysis. The resultant SNPs were C1096S, C545S, C664S, F1257L, F734S, I1174T, I1114T, P177S, P384S, W1198G, W1328G, W692C, W692L, W962R, Y499H. One functional domain of PLA2R1 is already modelled in PDB (6JLI), the full 3D structure of the protein was predicted using I-TASSER homology modelling tool. The stability analysis, structure superimposition, RMSD calculation and docking studies were carried out. The structural analysis predicted four mutations F734S, F1246L, I1174T, W1198G as damaging to the structure of the protein. All these mutations are occurring at the conserved region of CTL domain hence are more likely to abolish the function of the protein. Up to the best of our knowledge, this is the first study that provides in-depth and in-silico analysis of deleterious mutations on structure and function of PLA2R1.

## Introduction

Phospholipases play a vital role in many cellular processes including phospholipids digestion and metabolism. Phospholipases has four major types secretory phospholipase (sPLA2), cytosolic cPLA2, calcium independent iPLA2 and platelet activating factor (Lp) PLA2, are involved in lipid metabolism. In humans PLA2R1 encodes secretory phospholipases sPLA2 that acts as a receptor to bind various secretory phospholipases. Knockdown of PLA2R1 prevents senescence^1^.

PLA2R1 located on chromosome 2q23-q24 and is 180 kDa in weight is a transmembrane glycoprotein that acts as an endogenous ligand which stimulates the processes including cell proliferation and cell migration^2,3^. Phospholipases belongs to a group of enzymes that hydrolyzes the sn-2 ester of glycerol phospholipids that produces a fatty acid and a lysophospholipid. They are the enzymes that controls the release of lipid intervened precursors^4^. These phospholipases plays a key role in structure and cell signaling. Phospholipases which are activated by G-protein coupled receptors PLA2R1 results in releasing biologically active metabolite which further acts as first or second messengers to auxiliary modify or intensify the cell signals. Furthermore, it is also involved in many patho-physical situations varying from acute inflammatory conditions ending to cancer^5,6^. The PLA2 can provoke carcinogenesis, as the metabolite released which includes arachidonic acid is further metabolized into those molecules which induce cancer cell growth and proliferation. Cell cycle arrest is the permanent proliferative arrest and is activated by various stimuli including stress. This is on the whole is a tumor suppression mechanism that prevents from tumor progression. Failing the senescence will escort to tumor formation. PLA2R1 is a type I transmembrane glycoprotein receptor which acts as a regulator of senescence involving P53 pathway.

Current challenge for medical research is to associate the genetic risk factors with complex diseases. The most popular technique used so far is the genome wide association studies (GWAS) that associates variants with phenotypic traits. This involves the analysis of single nucleotide polymorphism (SNPs) refers to as single base change that is present in 1% of the whole population. One of the major relevance of studying SNPs is to comprehend the disease development associated with them. The major difficulty faced by the researchers is to develop a cost effective strategy to mine those functional SNPs from the millions of SNPs in a database^7^. SNPs are more frequently observed in the non-coding regions of the genome including 5′UTR and 3′UTR and introns. Those occurring at 5′UTR are involved in transcriptional activity while 3′UTR SNPs usually affects gene expression. The polymorphism occurring at intronic regions will affect the mRNA processing^8^. Coding SNPs in particular is getting attention among the researchers because the non-synonymous SNPs occurring in the coding regions of the genome are introducing the mutation at the amino acid level which in turn can be damaging to the structure or function of the protein. Damaging effects includes protein stability changes, protein–protein interactions and protein folding hence making them involved in various complex diseases^8,9^.

Several studies have shown the impact of missense mutations on the function of PLA2R1 and how it is associated with disease progression. The authors^10^ have reported the SNPs rs3749119, rs3749117, rs35771982, rs3828323, and rs4664308 as crucial for causing Nephropathy. Further^11^, have identified the two SNPs rs3749117 and rs35771982 that are causing protein level changes to the protein hence effecting its function. The experimental analysis carried out by^12^ showed that rs6757188 and rs3577198 are highly associated with idiopathic membranous nephropathy (IMN) and that’s maybe the underlying cause of IMN.

Numerous studies in the past have conducted various analysis particularly in-silico analysis that combines bioinformatics tool to predict the structural and functional consequences of nsSNPs occurring at the coding region of the genome. One study conducted SNP analysis in TAL1 gene which is a proto-oncogene hence is found associated with various hematological diseases^13^. Another study performed computational analysis on glycoprotein M6A that identified nsSNPs which are damaging to the structure and function of the protein^14^. The authors in^15^ identified the missense variants N391K and C414S in B-cell lymphoma leukemia 11A protein. A comprehensive study was carries out by^16^ to analyze the substitutions in TAGAP protein that combines computational tools of sequence, structure based. Additionally, the authors have added post translational modification analysis to more validate the findings^17^. One more study identified the SNPs in interleukin-8 (IL-8) gene for this purpose the study combines the structural functional analysis along with the docking studies to identify the effect of potential variants on protein–protein interactions^18^. Taking this into consideration, the present study aimed to explore the structural and functional impact of missense coding SNPs of PLA2R1 by using various computational tools. So far, no extensive in silico study has been reported on PLA2R1 that identifies the effect of SNPs exploring both sequence and structural features. To this end, this study conducted an in-depth analysis of PLA2R1 and its role and pathogenesis in complex diseases particularly cancer. In particular, the functions like protein stability and protein–protein interactions were explored in missense SNPs.

## Materials and methods

### Dataset retrieval

The dbSNP of NCBI was queried to obtain the human PLA2R1 SNPs hat has categorized the SNPs into 9 broad classes based on their location in the genome namely frameshift, inframe deletion, inframe indel, inframe insertion, initiator codon variant, intron, missense, non-coding transcript variant and synonymous (https://www.ncbi.nlm.nih.gov/snp/). The protein sequence of PLA2R1 was obtained from Uniprot (https://www.uniprot.org/) (UniProtKB—Q13018 (PLA2R_HUMAN)) and the protein structure file was obtained from RCSB PDB (PDB ID: 6JLI). Total of 29,537 SNPs were present out of which we have filtered 974 coding nonsynonymous/ missense SNPs for further analysis.

### Functional annotation of SNPs

All the retrieved SNPs were functionally annotated in order to find the most deleterious SNPs using 6 different tools. The first category of tools are sequence homology based which includes SNPNexus (https://www.snp-nexus.org/v4/) that has in-built SIFT and PolyPhen^19^, PROVEAN (https://provean.jcvi.org/index.php)^20^, and Mutation Accessor (https://mutationassessor.org/r3/)^21^. PolyPhen and SIFT categorizes the SNPs as damaging probably damaging or benign. Both the tools has different cut off thresholds and SNPnexus takes the average voting for categorizing a SNP as damaging or neutral. PROVEAN (Protein Variation Effect Analyzer) is a freely accessible webserver that takes in sequence of a protein and the list of variants to predict whether the mutations are affecting the function of the protein. The algorithm runs the homology search via blast and generates PROVEAN scores. The cutoff threshold is -2.5 as default which categorizes the mutations as neutral or deleterious. The mutation Accessor takes the variants in a particular format and predicts the functionally important ones by identifying the evolutionary conserved amino acid changes. The second category of the tools are consensus based methods which includes Meta-SNP (https://snps.biofold.org/meta-snp/index.html)^22^, SNPs&Go (https://snps.biofold.org/snps-and-go/index.html)^23^, and Predict-SNP (https://loschmidt.chemi.muni.cz/predictsnp/)^24^ webservers. The SNPs should be predicted deleterious from at least 4 out of 6 tools in order to be labelled as high risk SNP to avoid biasness in results.

### Identification of evolutionary conserved residues and motifs

The UniProt protein sequence of PLA2R1 was submitted to protein BLAST to run a homology search https://blast.ncbi.nlm.nih.gov/Blast.cgi. The 100 templates with a cutoff E-value below 1.00exp-20 and similarity threshold of > 30% were selected and submitted to CLUSTAL Omega (https://www.ebi.ac.uk/Tools/msa/clustalo/) for multiple sequence alignment. Further, the ConSurf^25^ webserver was utilized in order to analyze the SNPs occurring at conserved sites. The tool uses the Bayesian algorithm to calculate the conservation score by performing phylogenetics analysis between the homologous sequences. The MSA file generated from Clustal Omega^26^ was submitted as input to CONSURF and it generates the conservation profile labeled by coloring scheme and scores. The scores ranges from 1–4 as variable, 5–6 as intermediate and 7–9 as conserved. Further, the tool also predicts if the particular residue is buried or exposed which can further reveal the structural and functional importance of that residue. The tool is available at https://consurf.tau.ac.il/.

### Analyzing protein stability

To analyze the stability of the target protein we have I-Mutant server (https://gpcr2.biocomp.unibo.it/cgi/predictors/I-Mutant3.0/I-Mutant3.0.cgi)^27^ that is a support vector machine based approach that predicts the stability change of the protein upon mutation. It generates the reliability index ranging from 0 to 10 with 10 being the highest reliability. The PDB structure of PLA2R1 with position of variants were submitted as input with conditions of temperature 25 °C and pH 7.0. The tool provides the difference of Gibbs Free energy DDG value to determine the stability and de-stability of the protein structure upon mutation.

### Predicting disease related mutations using MutPred

To predict if the mutations are disease associated we have utilized two tools. MutPred (https://mutpred.mutdb.org/)^28^ classifies a variant as disease associated (pathogenic) or neutral by using three different in built tools namely Psi-BLAST, SIFT and PFAM which covers protein structure, function and evolution. It also includes structural disorder algorithms like TMHMM, MARCOIL, and DisProt hence, combining all these algorithms will bring a high confidence prediction score.

### 3D Structure modeling and effect of variants

The 3D structure of wild type PLA2R1 was obtained from PDB while the tertiary structure of mutant models were generated from I-TASSER homology modeling tool (https://zhanglab.ccmb.med.umich.edu/I-TASSER/)^29^. It combines sequence alignment with threading and ab-initio modelling and predicts the 3D structure in less time with no manual effort required. The resultant structures were further viewed by Chimera 1.11 which is an interactive visualization tool for molecular structures. The quality of the 3D model generated was also verified by ERRAT (https://servicesn.mbi.ucla.edu/ERRAT/?job=7462) which is a program used for validating the protein structures generated. Subsequently, TM-Align server (https://zhanglab.ccmb.med.umich.edu/TM-align/)^30^ was used to compare wild type and mutant structures which computes RMSD to analyze the deviation of mutants from the wild type.

### Pathway enrichment and molecular docking analysis

The STRING^31^ database (https://string-db.org/) was utilized to find the functional interacting partners of PLA2R1. Upon query it was observed that the closest binding partner was Mannose receptor 1 (MRC1). A high threshold of 0.7 was selected for generating this network. Next, the two proteins were docked by using ClusPro webserver^32^ https://cluspro.org/login.php by using default settings. The wildtype protein PLA2R1 with MRC1was docked along with the mutant models of PLA2R1-MRC1. For each experiment ClusPro predicts 10 different docked poses with the model scores that specifies the binding energy of the docked molecules.

## Results

### Dataset

**Figure 1:**
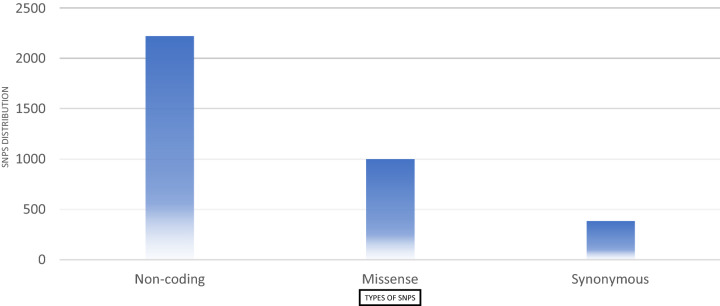
The frequency distribution of SNPs on different regions of the gnome.

**Figure 2:**
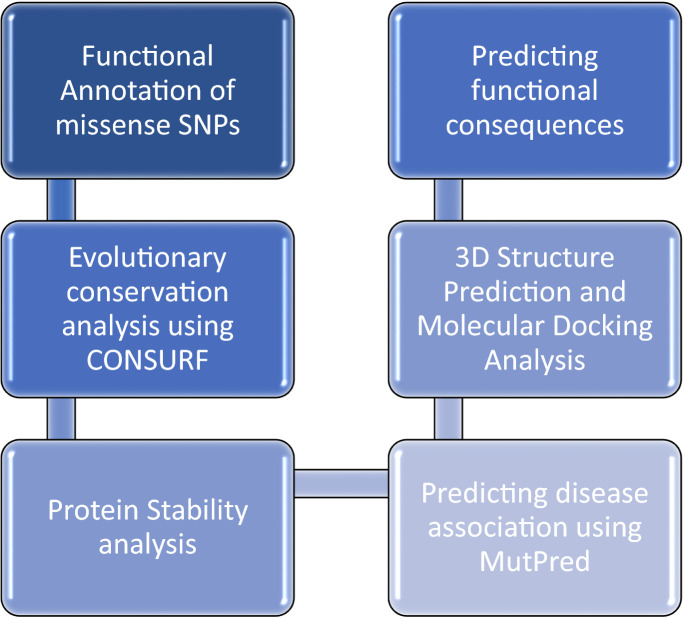
The overview of the workflow for identifying nsSNPs.

From dbSNP the total 974 missense SNPs were picked up for further analysis. These SNPs were selected based on the criteria that they should be sequenced in 1,000 Genomes Project and the SNPs should have minor allele frequency (MAF) data. The frequency distribution of the SNPs on synonymous, missense and non-coding genomes has been figured in Fig. 1. From the total 974 SNPs, SIFT and PolyPhen filtered 374 SNPs as damaging. These SNPs were further subjected to PROVEAN tool which further filters 220 SNPs as deleterious. Further, these SNPs were passed through 4 different tools namely Mutation Accessor, Meta-SNP, SNPs&Go, and Predict-SNP. We have labelled the SNPs as high risk only if they are predicted as damaging from at least 4 tools out of 6. The shortlisted high risk SNPs are tabulated in supplementary Table S1. A flowchart depicting the steps taken for carrying the analysis of current study is figured in Fig. 2.

### Conservation profile of SNPs

**Figure 3:**
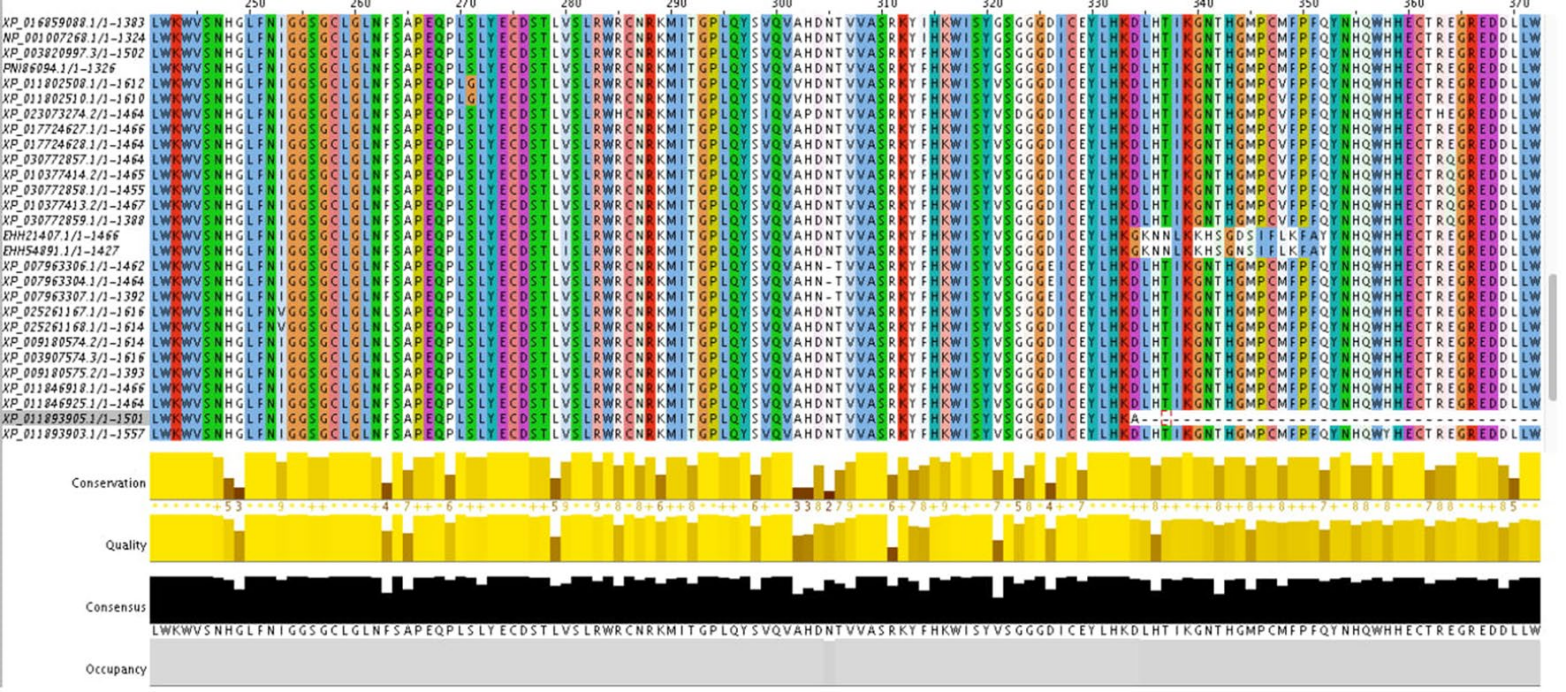
Multiple sequence alignment of PLA2R1 protein viewed in Jalview. The residues are colored using clustalW coloring scheme.

For determining the conservation profile of the selected SNPs we first have performed multiple sequence alignment using Clustal Omega. The output MSA has sent to CONSURF webserver to predict whether the SNPs are disrupting the conserved residues or are occurring at the variable regions. The variants occurring at positions 64, 73–77, 81, 83, 89–91, 106–107, 148, 159, 171–172, 267–269, 527, 531, 535, 577, 582, 701, 734, 838, 1,450, 1,174, 1,198, 1,455 are conserved residues present on core and surface of the protein hence, are more likely to effect the structure and function of PLA2R1. ConSurf uses the evolutionary conserved information along with the solvent accessibility data to explore the structurally and functionally important residues. The detailed results are tabulated in Table S2. The multiple sequence alignment is shown in Fig. 3 which is viewed in Jalview version1.8.3-1.1.8 and can be downloaded from https://www.jalview.org/getdown/release/.

### Validating disease associated SNPs using MutPred

The high confidence SNPs were further subjected to MutPred to analyze if these SNPs are associated with disease. MutPred predicts the molecular mechanisms associated with the SNPs along with that it also predicts if the mutations are creating any gain or loss of catalytic style, solvent accessibility, and posttranslational modifications. MutPred scores g value > 0.75 and the p-value < 0.05 are observed as confident hypothesis hence, we selected high risk SNPs based on this cutoff. The results are tabulated in Table S3. The high scored mutations are W1198G, I774T, F734S, and W692C. These SNPs were also predicted as damaging from the tools SNPNexus, PROVEAN, Meta-SNP, PHDSNP and SNPs&GO (Table 1).

**Table 1:** High risk mutants predicted from 8 tools.

Mutation	Score	Effect
C1096S	− 2.083	Highly destabilizing
C545S	− 2.176	Highly destabilizing
C664S	− 2.32	Highly destabilizing
F1257L	− 2.121	Highly destabilizing
F734S	− 3.067	Highly destabilizing
I1174T	− 2.213	Highly Destabilizing
P1114T	− 2.364	Highly destabilizing
P177S	− 2.295	Highly destabilizing
P384S	− 2.376	Highly destabilizing
W1198G	− 3.492	Highly destabilizing
W1328G	− 2.999	Highly destabilizing
keggW692C	− 2.061	Highly destabilizing
W692L	− 2.825	Highly destabilizing
W962R	− 2.228	Highly destabilizing
Y499H	− 2.323	Highly destabilizing

### Protein stability change prediction

Protein stability alterations were predicted using I-Mutant, the shortlisted high risk SNPs were submitted to predict the change in Gibbs free energy. The results revealed that out of all, there are 15 mutants C1096S, C545S, C664S, F1257L, F734S, I1174T, P177S, P384S, W1198G, W1328G, W692C, W692L, W962R, Y499H that were predicted as highly destabilizing hence are expected to cause maximum damage to the protein by affecting its stability. The results are tabulated in supplementary Table S1.

### 3D modelling of wild type PLA2R1 and its mutants with functional characterization using pathway analysis

**Figure 4:**
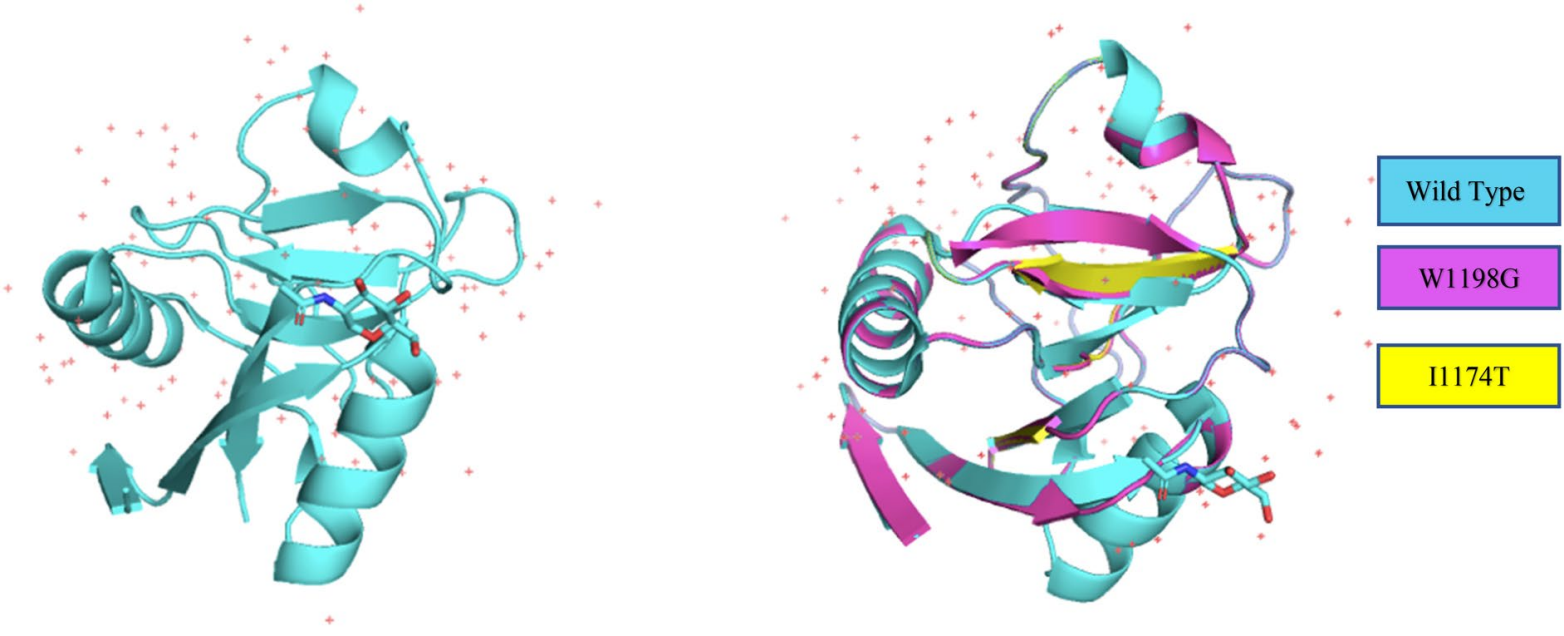
The crystal structure of CTLD domain of PLA2R1 (1,108–1,234) with NAG Inhibitor with superimposed mutant models of W1198G and I1174T.

**Figure 5:**
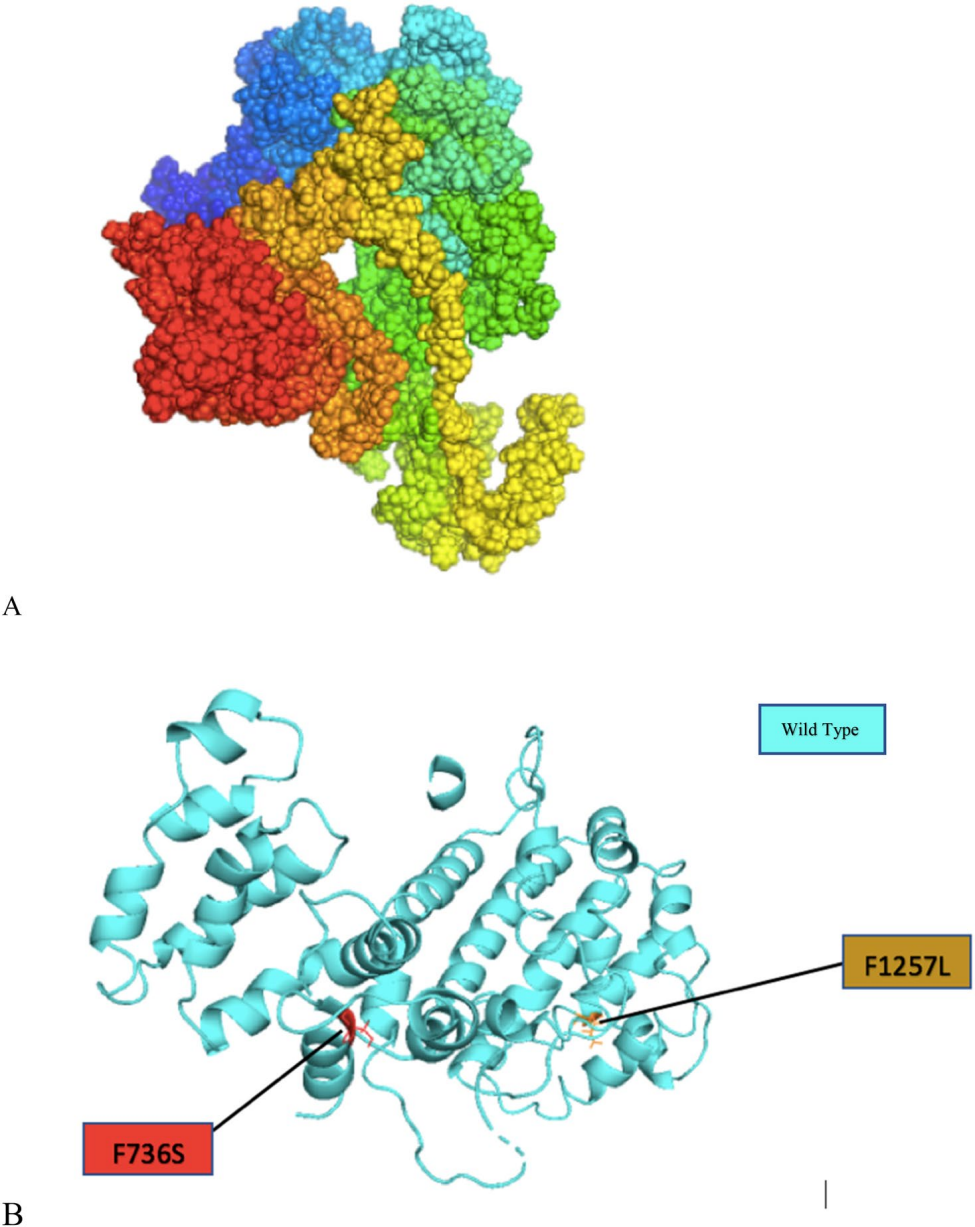
Comparison of wild type with its mutant (**A**) is 3D model of PLA2R1 and (**B**) shows the superimposed model of wildtype PLA2R1 with F734S and F1257L mutants.

The 3D crystal structure of CTLD domain of PLA2R1 was already present in RCSB PDB which have residues modelled from 1,108 to 1,234. We have determined the full tertiary structure by using homology modelling server I-TASSER. Further, to determine the structural effect of the high risk SNPs, the mutant models were also generated using I-TASSER. Moreover, these structures were subjected to TM-Align for computing RMSD to analyze the variation of wild type and mutants. The higher RMSD indicates the higher deviation of mutant from its wild type. Based on the RMSD values obtained, from the crystal structure of CTLD domain of PLA2R the mutants W1198G and I1174T showed the deviation of 8.25 and 7.36 while the mutant models of F1257L and F734S in fully modeled 3D structure showed the maximum RMSD of 10 and 10.2 hence, we have selected only these mutants to remodel them using I-TASSER in order to generate the reliable structures. Further, these mutants were also superimposed with the wild type protein structure. The quality of the predicted models were also verified by using ERRAT which showed the quality factor of 73.25 for the wild type I-TASSER generated model, 79.577 for F1257L and 79.63 for F734S. The mutations are figured in Figs. 4 and 5.

**Figure 6:**
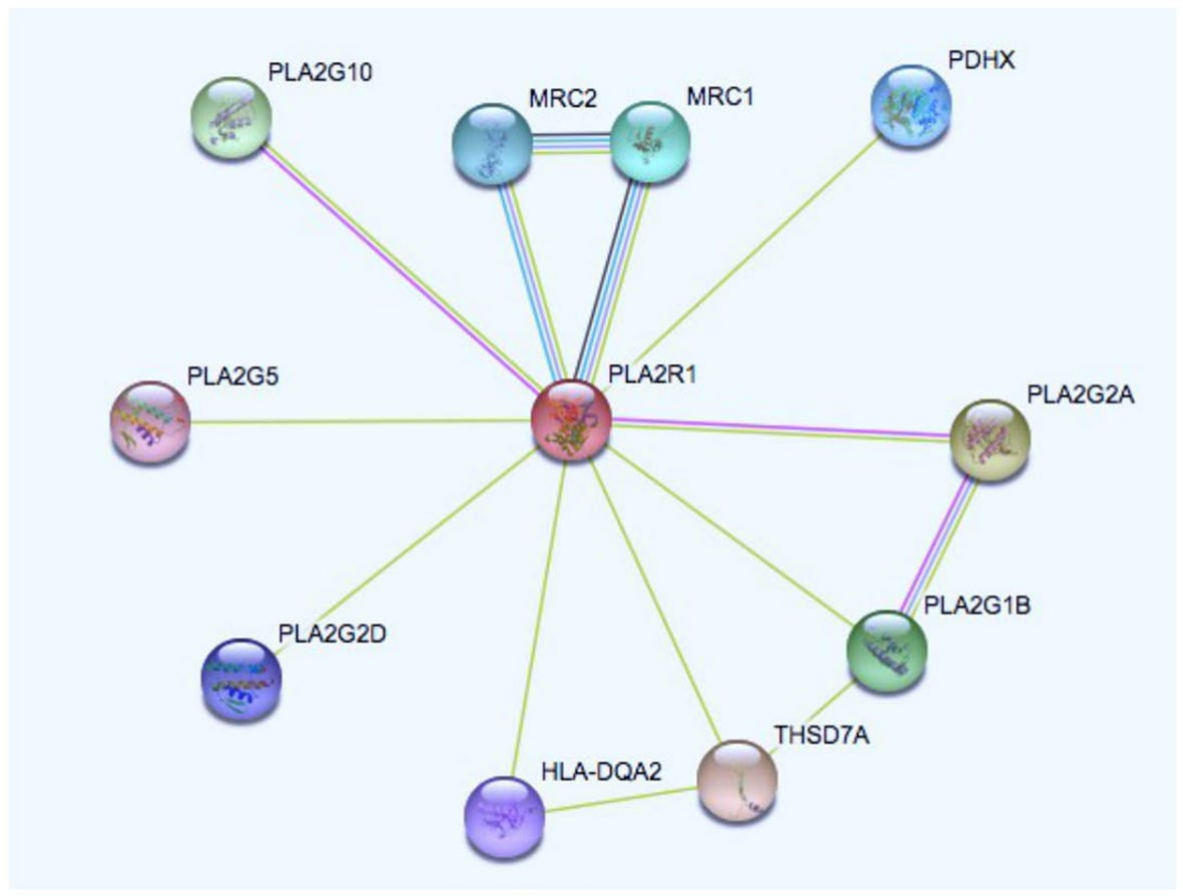
The pathway enrichment analysis carried out by STRING database that determines the interacting partners of PLA2R1 categorized on the basis of interacting type.

**Figure 7:**
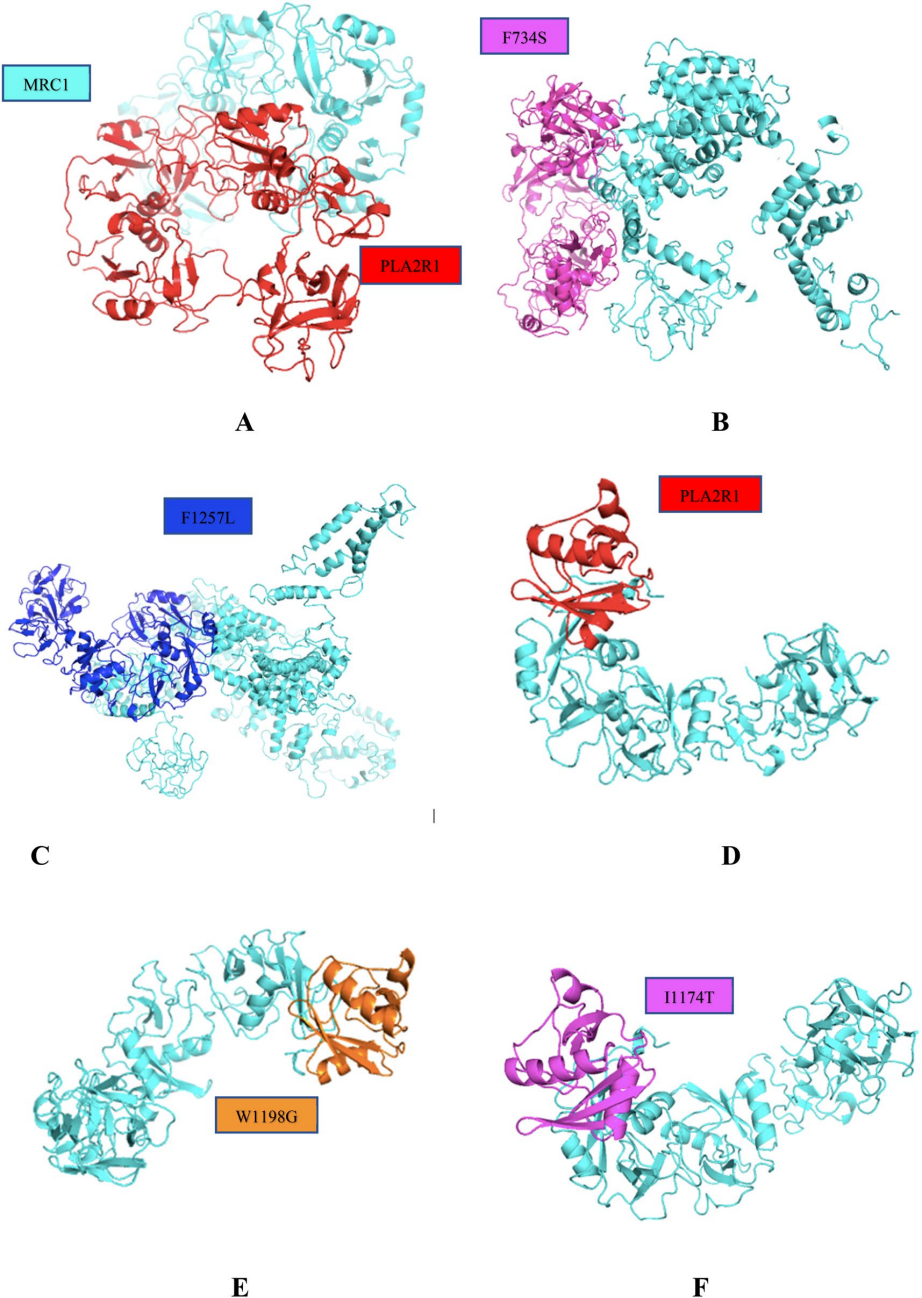
(**A**) shows the wildtype PLA2R1 interaction with MRC1. (**B**, **C**) shows the mutations affected interaction of the two proteins. (**D**–**F**) shows the docked poses of 6JLI-5XTS PDB proteins and mutations of PLA2R1 W1198 and I1174T.

The protein–protein interaction network was generated using STRINGS database that predicts the interactions based on co-gene expression, physical interactions, binding etc. The PPI enrichment value was < 1.0e−16 with average clustering coefficient is 0.689. The KEGG pathways^33^ reported are tabulated in Table 2 and the pathway is figured in Fig. 6. Further, the highly interacting partner of PLA2R1 was MRC1 and the interacting type is binding hence, we first predicted the 3D structure of MRC1 using I-TASSER and then used ClusPro to conduct docking studies on PLA2R1 and MRC1. The crystal structure of MRC1 was present in PDB (5XTS) with few residues modelled from 29–629 however, the sequence length of MRC1 is 1,455 amino acids. We used both structures for molecular docking. Phospholipase A2 is a receptor for secretary lipases which by binding regulates the activation of the mitogen-activated protein kinase (MAPK) cascade to induce cell proliferation. Mannose receptor 1 on the other hand, mediates the endocytosis of glycoproteins by macrophages and it binds both sulfated and non-sulfated polysaccharide chains^34^. We carried out docking with wildtype PLA2R1-MRC1 and also with the mutants PLA2R1-MRC1 to check if the mutations are affecting the molecular interactions between the two proteins. ClusPro is a webserver for direct docking of two interacting proteins. On providing the two interacting proteins as input the server rotates the ligand with 70,000 rotations first and then picked 1,000 rotations with the lowest score. After that, using greedy approach 1,000 ligand positions were clustered with a 9oA C-alpha RMSD radius hence generating its neighbors. The Fig. 7 shows the docked poses of wild type and mutants PLA2R computationally predicted structure with MRC1 and also the crystal structure of CTL domain of PLA2R with crystal structure of MRC1. There are four modes of docking namely Electrostatic favored, hydrophobic favored, vanderwals + electrostatic and balanced. We have selected balanced mode. The models are ranked based on their lowest energy. The mutation is occurring at the conserved region and is located in a domain. In addition to that , in order to measure the strength of interaction between the two proteins we have used another measure i-e buried surface area (BSA) of the wildtype docked complex and the mutant docked complex of the two proteins. Higher value indicates the more stable structure so, the computed BSA of the wildtype docked molecules shows that it is more stable as compared to the mutant models. The computed BSA is tabulated in Table 3.

**Table 2:** List of KEGG pathways.

Pathway	Description	Count in gene set
Hsa04975	Fat digestion and absorption	3 of 39
Hsa04145	Phagosome	4 of 145
Hsa00592	Alpha-Linolenic acid metabolism	3 of 25
Hsa05152	Tuberculosis	4 of 172

**Table 3:** Buried surface area of the wildtype bound complex and the mutant bound complex.

Protein bound complex	Buried SA (A^2^)
PLA2R1-MRC1 (Wildtype)	4,920
PLA2R1 (F734S)-MRC1	3,432
PLA2R1 (F1257L)-MRC1	4,026
CTLD PLA2R1-MRC1 (Wildtype)	1,880
CTLD PLA2R1 (W1198G)-MRC1	1785
CTLD PLA2R1 (I1174T)-MRC1	1782

For the mutations I1174T and F1257L HOPE webserver (https://www3.cmbi.umcn.nl/hope/) predicted that these two mutations are occurring at domain c-type lectin 7. The wild type residues are highly conserved and more hydrophobic in nature then the mutant residues which eventually leads to loss of protein–protein interactions. The mutation F734S is also occurring in the conserved region as the previous two mutations. Further, the wild-type residue Phenylalanine is involved in cysteine bridge formation as annotated in UniProt, which is important for stability of the protein. The mutation occurring at this position will cause loss of interaction and ultimately disrupt the structure of the protein. Altogether, the loss of the cysteine bridge formation and the difference of wild type and mutant protein causes protein destabilization. The mutation W1198G is also occurring at CTL domain, the mutation replaces Tryptophan with Glycine at this position. Glycine are very flexible amino acid hence can disturb the rigidity of the protein required at this particular position^35^.

## Discussion

Single nucleotide polymorphisms (SNPs) are referred as the most common genetic variants that are associated with various complex diseases. The SNPs occurring at the coding regions of the genome tend to be more damaging to the structure and function of the protein hence, are the most studied in the current research. Currently, medical research is more focused in analyzing the impact of these deleterious non-synonymous SNPs that also have implications in various complex diseases. The studies focused on determining the functional consequences of these SNPs by predicting if the particular SNP has neutral effect or is disease causing by exploiting both sequence and structural features.

In this study, the comprehensive analysis of nsSNPs in PLA2R were explored. To improve the prediction accuracy we have combined tools from different categories namely sequence based, homology based, consensus based and structure based. This approach will provides high confidence in selecting potentially damaging SNPs by avoiding the biasness in the results. The SNPs were first annotated with 6 different bioinformatics tools namely SNPNexus (SIFT- POLYPHEN), PROVEAN, MetaSNP, PHDSNP and SNPs&Go that categorizes the disease associated SNPs with the neutral ones. The pre-filtered SNPs were further subjected to structure–function analysis. The evolutionary conservation analysis carried out by CONSURF predicted few structurally conserved residues that were further evaluated for protein stability analysis. The 3D structure of one of the domains CTLD in PLA2R is already deposited in PDB. For the rest of the structure we have utilized I-TASSER homology modelling tool and further refined and energy minimized by TM-Align. The structural impact of these mutations were checked by mapping the mutations onto the 3D structure of the protein in other words the wild type and mutant models were superimposed using PyMOL. Structural changes can be viewed by analyzing either the stability change in the protein or by analyzing the conformational change which further effects the binding of the protein. The high risk SNPs after protein stability analysis were C1096S, C545S, C664S, F1257L, F734S, I1174T, I1114T, P177S, P384S, W1198G, W1328G, W692C, W692L, W962R, Y499H as predicted the destabilizers. Next step was to check if they are changing the conformation of the protein, we first run minimization to the mutant structures and visualized the RMSD for them. The RMSD scores generated showed that the W1198G, I1174T, F1257L and F734S in I-TASSER generated model were highly deviated as these mutants were also predicted as high destabilizers hence, we picked these mutants for carrying out the docking analysis.

From the docking analysis carried out by ClusPro, showed that the mutations are affecting the interaction of PLA2R and MRC1 which was necessary for initiating cell proliferation. PLA2R protein structure has extracellular domains, a transmembrane domain and a short cytoplasmic tail^34^. It has a functional c-type lectin domain which is involved in various cellular process like receptor binding, immunity and angiogenesis. All the high risk mutations are occurring at c-type lectin domain, C-type stands for calcium binding, proteins that have these domains are involved in variety of cellular processes including cell–cell adhesion, immune response and apoptosis. This conserved domain functions as carbohydrate binding domain usually its 110–130 residues long also there are four cysteines that are conserved also involved in forming disulfide bridges. The structure of CTLD contains a double loop also called as loop in a loop which is stabilized by these disulfide bridges. This structural loop is very flexible in nature and also involved in carbohydrate binding. Hence, mutation in this domain can abolish the function of the protein. The four high risk SNPs are occurring at CTL domain and out of them the F1257S is involved in disulfide bridge formation thus, this mutation might be more damaging to the function of the protein hence leads to loss of interactions. The interacting energy for the wild type PLA2R1-MRC1 interaction was − 241 and 671.9 for the docked PDB structures of both proteins. Upon calculating their mutants docked structures with MRC1, a huge decrease of binding energy found which was − 1,268.2 for F1257L and F734S and for W1198G − 839.4, I1174T was 870.9. These scores were calculated by combining all the energy values from Vanderwal, Electostatic and Hydrophobic. Thus the difference in docking energies upon mutation is disrupting the normal activity of the protein. Also, the buried surface area showed that the wildtype docked complex is more stable as compared to the mutant models, hence mutations are effectively disrupting the interaction between the two proteins.

In this study, we have determined high risk SNPs in PLA2R1 by using combination of different bioinformatics tools. The results have determined W1198G, I1174T, F1257L and F734S as the most damaging nsSNPs having both sequential and structural consequences. Our study provides an in-depth analysis of missense variants in PLA2R1which can further be verified by experiential analysis to determine its role more precisely. The mutations caused by these SNPs have functional consequences as predicted above hence, they are more likely to initiate disease formation.

## Supplementary information

Supplementary Table S1.

Supplementary Table S2.

Supplementary Table S3.
